# Resveratrol-Loaded Polymeric Nanoparticles Protect Against Rotenone-Induced Parkinsonian-Like Cellular Damage In Vitro: Association with NRF2/HMOX-1 Expression Changes

**DOI:** 10.1007/s11064-026-04749-z

**Published:** 2026-04-13

**Authors:** Izabell Maria Martins Teixeira, Bruna Ribeiro Duque, Mac Dionys Rodrigues da Costa, Mateus Edson da Silva, Mateus Oliveira Fernandes, Antônia Gabriella de Souza Freitas, Vitor Mendes Bezerra, Alice Vitória Frota Reis, Natasha Maria Lima Pinheiro, Marco Antonio de Freitas Clementino, Josimar Oliveira Eloy, Ramon Róseo Paula Pessoa Bezerra de Menenzes, Alice Maria Costa Martins, Tiago Lima Sampaio

**Affiliations:** 1 Department of Clinical and Toxicological Analysis, Federal University of Ceará, Pastor Samuel Munguba Street, 1210, Fortaleza, 60430372 Ceará Brazil; 2 Department of the Physiology and Pharmacology, Federal University of Ceará, Coronel Nunes de Melo Street, 1127, Fortaleza, 60430275 Ceará Brazil; 3 Department of Pharmacy, Federal University of Ceará, Pastor Samuel Munguba Street, 1210, Fortaleza, 60430372 Ceará Brazil

**Keywords:** Nanotechnology, Resveratrol, NRF2 signaling pathway, Antioxidant, Neurodegenerative diseases

## Abstract

Parkinson’s disease (PD) is a progressive neurodegenerative disorder with limited treatment options. Several natural compounds have been investigated, particularly resveratrol (RSV), which exhibits antioxidant and anti-inflammatory properties. However, its unfavorable pharmacokinetic profile limits its therapeutic application, making nanoencapsulation a promising strategy. This study evaluated the protective effects of resveratrol-loaded polymeric nanoparticles (NP RSV) and the involvement of the Keap1/NRF2/ARE pathway in a rotenone (ROT)-induced PD-like model in vitro. PC12 neuronal cells and astrocytes were pretreated with NP RSV, RSV, and dopamine for 1 h, followed by ROT exposure for 24 h. Cell viability was assessed by MTT, while cell death profile, reactive oxygen species production, and mitochondrial transmembrane potential (ΔΨm) were evaluated by flow cytometry. Morphological changes were evaluated by optical microscopy. Gene expression of *NRF2* and heme oxygenase-1 (HMOX-1) was assessed by RT-qPCR. Pretreatment with NP RSV significantly protected cells by preserving viability, reducing reactive oxygen species, maintaining mitochondrial function, and decreasing apoptosis. Morphological analyses corroborated these results. Furthermore, NP RSV modulated ROT-induced *NRF2* and *HMOX-1* expression, suggesting involvement of the Keap1/NRF2/ARE pathway.

**Supplementary Information:**

The online version contains supplementary material available at 10.1007/s11064-026-04749-z.

## Introduction

 Parkinson’s disease (PD) is a progressive neurodegenerative disorder characterized by the accumulation of misfolded alpha-synuclein (α-Syn), forming Lewy bodies in the substantia nigra. This process results in the loss of dopaminergic neurons and the emergence of motor and non-motor symptoms, such as resting tremor, bradykinesia, muscle rigidity, neuropsychiatric changes, and autonomic disorders [1]. It is the second most prevalent neurodegenerative disease in the world, predominantly affecting elderly males, and its prevalence is likely to increase significantly in the coming decades as a result of population aging [2–4]. It has significant social and economic impacts on healthcare systems as well as on patients and their families. Studies show that annual costs average €20,911.37 per patient [5].

The pathophysiology of PD is multifactorial and involves complex mechanisms, notably oxidative stress and neuroinflammation, which are directly associated with neuronal degeneration and glial cell activation, compromising essential components of the neuroimmune system [6]. Thus, changes in pathways related to the balance between reactive oxygen species (ROS) production and brain antioxidant capacity have been extensively investigated, including excessive ROS production and dysfunction of Nuclear Factor Erythroid 2-Related Factor 2 (NRF2) [1, 7].

NRF2 plays a central role in cellular defense against oxidative and inflammatory damage. Under basal conditions, this transcription factor is associated with the repressor protein Keap1 (Kelch-like ECH-associated protein 1) in the cytoplasm, but under oxidative stress, it dissociates from this protein, binding to short DNA sequences known as Antioxidant Response Elements (ARE) and promoting the expression of antioxidant systems including reduced glutathione (GSH), superoxide dismutase (SOD), and heme oxygenase 1 (HMOX-1) [8]. In this sense, naturally occurring bioactive molecules, such as resveratrol (RSV), are described as modulators of this pathway, capable of stimulating it and exerting relevant cytoprotective effects [9].

Despite advances in understanding these molecular mechanisms, the treatment of PD remains essentially symptomatic, based mainly on dopaminergic replacement with drugs such as levodopa (l-Dopa), which loses efficacy and has adverse motor effects when used chronically, without preventing the progression of the disease [10]. In this scenario, RSV stands out for its antioxidant, anti-inflammatory, and neuroprotective properties, showing promising effects in experimental models of neurodegenerative diseases by attenuating initial events associated with neurodegeneration [11, 12]. However, its clinical application is limited by low bioavailability, chemical instability, short half-life, and rapid metabolism [13].

Given these limitations, the development of resveratrol polymeric nanoparticles (NP RSV) based on poly-ε-caprolactone (PCL) polymer and D-α-tocopheryl polyethylene glycol 1000 succinate (TPGS) emerges as a promising strategy to optimize resveratrol delivery, improving its stability, bioavailability, and tissue distribution while enabling controlled release and reducing adverse effects [14, 15].

PCL-based polymeric NP have been studied as drug carriers, including anticancer agents, antibiotics, and anti-inflammatory drugs [16, 17]. This formulation offers several advantages over lipid-based and inorganic nanoparticles. PCL is resistant to chemical hydrolysis, presents high drug permeability, improves solubility and drug delivery, enables controlled drug release, and is biodegradable. In addition, it shows favorable delivery performance to the central nervous system (CNS) [18, 19]. In addition, TPGS has already been reported in the literature as an important factor in prolonging drug circulation, improving the release of the encapsulated drug, and increasing encapsulation efficiency, as well as reducing drug resistance [20–22]. Although research has advanced, there are currently no clinically approved nanoencapsulated therapies specifically for PD., despite the clear advantages of this formulation.

Considering the high prevalence of PD, its complex pathophysiology, and the absence of therapies capable of modifying the course of the disease [23], it is important to investigate innovative therapeutic strategies. In this context, the present study aims to evaluate the cytoprotective effect of resveratrol-loaded polymeric nanoparticles (NP RSV) and their involvement in the modulation of the Keap1/NRF2/ARE pathway in an in vitro rotenone (ROT)-induced model of Parkinson’s disease (PD). In this way, the study seeks to contribute to the elucidation of neuroprotective mechanisms and to provide insights for the development of more effective and safer therapeutic approaches that go beyond exclusively symptomatic treatment.

## Materials and Methods

### Source of Substances

The following substances were used in the study: resveratrol (RSV), dopamine (DA), and ROT. All chemicals were obtained from Sigma-Aldrich (St. Louis, MO, USA). Resveratrol polymeric nanoparticles (NP RSV) were obtained in partnership with the CEDEFAR-GPNANO laboratory at the Federal University of Ceará (Fortaleza, CE, BRA) according to previously standardized methods [15].

### Preparation and Characterization of Nanoparticles

NP RSV were prepared by the nanoprecipitation method using poly(ε-caprolactone) (PCL) as the polymer matrix and D-α-tocopheryl polyethylene glycol 1000 succinate (TPGS) as a surfactant, as originally described by our previous study [15], where detailed physicochemical characterization was reported. PCL exhibits physicochemical characteristics that make it suitable for nanoparticle production and drug delivery, while TPGS improves drug release and encapsulation efficiency. Both nanoparticles containing the active compound (NP RSV) and blank nanoparticles (NPB) were characterized in terms of particle size, polydispersity index (PDI), and zeta potential by dynamic light scattering (DLS) using a ZetaSizer Nano ZS (Malvern Instruments, United Kingdom), with a laser beam operating at 10 mV He-Ne, a wavelength of 633 nm, an incidence angle of 175°, and a temperature of 25 °C. For this purpose, the formulations were diluted in ultrapure water (1:10) and analyzed in triplicate following the manufacturer’s instructions, and the results were expressed as mean ± standard deviation (SD).

### Cell Culture

The cell lines used were transplantable rat pheochromocytoma, PC12 (APABCAM, Rio de Janeiro, RJ, BRA) and murine astrocytes, isolated and supplied by the Federal University of São Paulo (UNIFESP) [24].

For the experiments, when the cells reached confluence, they were detached (0.05% trypsin in 0.53 mM ethylenediaminetetraacetic acid - EDTA) and plated in 6-well (3 × 10^5^ cells/mL), 24-well, and 96-well plates (1 × 10^5^ cells/mL) in Dulbecco’s Modified Eagle’s Medium (DMEM) supplemented with 10% fetal bovine serum (FBS) and antibiotics (penicillin—200 IU/mL and streptomycin—130 µg/mL).

### Experimental Design

To evaluate the potential of RPNs in protecting against ROT-induced PD-like cell damage in vitro, an experimental protocol was proposed after verifying the cytotoxicity of the substances individually and the IC50 (half-maximal inhibitory concentration) of ROT. For this purpose, screening assays were performed at decreasing concentrations of NP RSV (PC12: 0.39–100 µM; AST: 1.56–100 µM), RSV (1.56–100 µM), and DA (12.5–800 µM) to determine the non-toxic concentration range applicable to cytoprotection. These concentrations were used in all subsequent experiments. Subsequently, cells were pretreated with the substances for 1 h, followed by ROT exposure for 24 h to induce cellular damage.

The selected concentrations were evaluated for their cellular protection mechanisms compared to the negative control, containing only cells, and the ROT group. Dopamine (DA) was used as a pharmacological control, considering l-Dopa as the standard dopaminergic therapy, which is a prodrug that is converted into dopamine in the brain. Three independent experiments were conducted, all in triplicate (*n* = 3).

The dilutions were made in sterile phosphate-buffered saline (PBS, consisting of 137 mM NaCl, 2.7 mM KCl, 1.47 mM KH₂PO₄, and 8.1 mM Na_2_HPO_4_; pH 7.4), using dimethyl sulfoxide (DMSO) as a co-solvent to facilitate solubilization, so that the final concentration of DMSO in the experiments did not exceed 0.5%, a concentration considered non-toxic for cell lines [25].

### Cell Viability Assay

The cells were subjected to the treatment described above with different concentrations of RPN. Cell viability was assessed by the MTT assay based on the reduction of 3-(4,5-dimethylthiazol-2-yl)-2,5-diphenyltetrazolium bromide (MTT) to insoluble formazan crystals by metabolically active cells [26, 27].

In this assay, the cells were kept in 96-well plates overnight. After that, the substances were added and incubated for 1 h to be exposed to ROT and kept for 24 h after which the medium was removed and pure DMSO was added to promote the formation of formazan from cell lysis. Finally, the plate was shaken for 30 min, and the absorbance was read in a microplate reader at a wavelength of 595 nm, and the percentage of cell viability, which is proportional to the absorbance, was calculated.

### Flow Cytometry Assays

For this assay, cells were cultured in 24-well plates at a minimum concentration of 1 × 10^5^ cells/mL. They were then washed with sterile phosphate-buffered saline (PBS, consisting of 137 mM NaCl; 2.7 mM KCl; 1.47 mM KH_2_PO_4_; 8.1 mM Na_2_HPO_4_ and pH 7.4), trypsinized, and centrifuged. To enable fluorescent labeling, the supernatant was replaced with a binding buffer, also at pH 7.4, consisting of 140 mM NaCl, 2.5 mM CaCl₂, and 10 mM HEPES. Finally, all cells were analyzed on FACSCalibur equipment (BD Biosciences, New Jersey, USA) using Cell Quest Pro™ software.

### Evaluation of the Cell Death Pathways (7-AAD/Anx)

The molecules 7-amino-actinomycin (7-AAD) and Annexin-V-PE (Anx) were used as markers to evaluate cell death pathways. 7-AAD is a derivative of actinomycin D, with excitation at 488 nm and fluorescence emission at 647 nm, which selectively binds DNA and penetrates only cells with compromised membrane integrity. Therefore, increased labeling of cells with this fluorochrome signals loss of cell membrane integrity, a characteristic commonly observed in necrosis processes [28].

Annexin V binds phosphatidylserine exposed on the outer leaflet of the plasma membrane during early apoptosis. Phosphatidylserine is a membrane phospholipid that is externalized when there is signaling of programmed cell death by apoptosis. Therefore, cells that show high labeling with this molecule are considered apoptotic. In addition, some cells may exhibit both markers, a process characteristic of late apoptosis, in which membrane integrity was lost and only then did programmed cell death occur [29].

### Evaluation of Cytoplasmic Production of Reactive Oxygen Species

For this assay, 2′,7′-dichlorofluorescein diacetate (DCFH-DA) was used. This is a lipophilic and non-fluorescent molecule, properties that make it easier to cross the plasma membrane. Some cytosolic enzymes have the ability to promote the deacetylation of DCFH-DA to form 2′,7′-dichlorofluorescein (DCFH), which, due to its high polarity, is retained in the cytoplasm, where the ROS produced oxidize DCFH to DCF (oxidized), which exhibits green fluorescence [30].

To this, the cells were treated with the substances, and after 3 h, a 20 µM DCFH-DA solution was added. After 24 h, the cells were trypsinized, centrifuged, washed in PBS, and resuspended in 500 µL of saline solution for analysis by flow cytometry. The intensity of green fluorescence (515 to 545 nm) was measured, and the experimental groups were compared by calculating the Relative Fluorescence Intensity (RFI) according to the equation:

$$RFI = \text{Fluorescence \;of\; the \;treated \;group/Fluorescence\; of\; the \;control \;group}$$

### Assessment of Mitochondrial Transmembrane Potential (ΔΨm)

To assess mitochondrial damage, mitochondrial transmembrane potential (ΔΨm) was evaluated by labeling with the cationic fluorochrome Rhodamine 123 (Rho 123). This fluorochrome is strongly attracted to the negative electrical potential of the mitochondrial membrane, which is reduced when there is damage to the membrane of this organelle, as is the labeling with this fluorochrome [31–34]. The cells in the control group, treated with ROT or treated with different concentrations of the substances, were labeled with Rho 123 at a concentration of 10 µg/mL 30 min before reading in a flow cytometer.

### Optical Microscopy

For optical microscopy analysis, the cells were viewed under a Nikon^®^ inverted microscope (Eclipse Ti) at 100× magnification. The Infinity Analyze software was used for this purpose. The morphological characteristics and cell density of each group were analyzed.

### Gene Expression Analysis

Molecular analyses were performed to analyze changes in the expression of *NRF2* and HMOX-1 genes. For this purpose, total RNA was extracted, followed by enzymatic digestion of DNA fragments using the Fast RNA extraction with PureLink RNA Mini Kit (Thermofisher). The entire extraction protocol was performed under refrigeration, in which the process was controlled by determining the concentration and purity of RNA and DNA in a Nanodrop™ microvolume spectrophotometer (Thermo Fisher^®^), aiming at the analysis of RNA purity and concentration in ng/µL (CFX96 Touch Real-Time PCR Detection System – Bio Rad).

Complementary DNA synthesis was then performed by reverse transcriptase reaction, in which the expression of the genes of interest was evaluated by Reverse Transcription quantitative Polymerase Chain Reaction (RT-qPCR) using the reaction mix (SYBR Green Master Mix - NZYtech) and specific sense and antisense primers: *NRF2* (forward—ATCTCCTAGTTCTCCGCTGCT; reverse—CTCCAAGTCCATCATGCTGAGG); *HMOX-1* (forward—GCCACCAAGGAGGTACACAT; reverse—GCTTGTTGCGCTCTATCTCC); *GAPDH* (forward—GCTTGTTGCGCTCTATCTCC; reverse—GAGTTGCTGTTGAAGTCGCA) [35, 36]. Glyceraldehyde-3-phosphate dehydrogenase (GAPDH) was used as a housekeeping gene [37, 38].

For quantification, analytical curves were created using serial dilutions of a standard sample. The slope of this standard curve was used to calculate the efficiency of the primers. In the data analysis, the 2^−ΔΔCt^ method was used for relative quantification, comparing the gene of interest with the reference gene [35].

Melting curve analysis was performed at the end of each real-time PCR run using a gradual increase in temperature according to the instrument protocol. This procedure was used to assess the specificity of amplification by verifying the presence of a single, well-defined melting peak, indicating a homogeneous PCR product and the absence of nonspecific amplification or primer-dimer formation. Only reactions that met these quality criteria were included in the subsequent analyses.

### Statistical Analysis

Statistical analysis of the data was performed using GraphPad Prism version 8.0 for Windows (GraphPad Software^®^, San Diego, California, USA). Normality was tested using the Shapiro-Wilk test. Subsequently, the analysis of variance test (one-way and two-way ANOVA) was applied, followed by the Bonferroni test (post hoc) for parametric results. All data were expressed as mean ± SD, considering values significant when *p* < 0.05.

## Results

### Characterization of Nanoparticles

RSV-loaded and blank nanoparticles were characterized in terms of particle size, polydispersity index (PDI), and zeta potential, and the obtained results are presented in Table 1. For both nanocarriers, particle sizes below 200 nm and polydispersity indices equal to or lower than 0.3 were obtained, in agreement with our previously published study, indicating adequate reproducibility of the nanoparticle preparation method. In addition, as demonstrated in detail in our previously published work, the NP RSV explored here showed drug encapsulation efficiencies above 90% and released percentages close to 50% after 50 h under experimental conditions that mimic the physiological environment.

**Table 1:** Characterization of nanoparticles

Formulation	PDI	Diameter ± SD (nm)	ZP (mV)
NP RSV	0.379	169.4 ± 72.4	− 5.8 ± 1.22
NPB	0.260	123.2 ± 73.16	− 4.2 ± 1.25

*NP* Nanoparticle, *NP RSV* resveratrol polymeric nanoparticles, *NPB* blank nanoparticles, *PDI* polydispersity index, *ZP* zeta potential

The substances were tested for cytotoxicity in cell lines. For this purpose, non-toxic concentrations for PC12 were selected: NP RSV (1.56 µM, 0.78 µM, and 0.39 µM); RSV (12.5 µM, 6.25 µM, and 3.12 µM); DA (400 µM, 200 µM, and 100 µM), and for AST: NP RSV (6.25 µM, 3.12 µM, and 1.56 µM); RSV (12.5 µM, 6.25 µM, and 3.12 µM); DA (400 µM, 200 µM, and 100 µM), as shown in the supplementary material (Fig. 9 in Supplementary material).

**Fig. 1:**
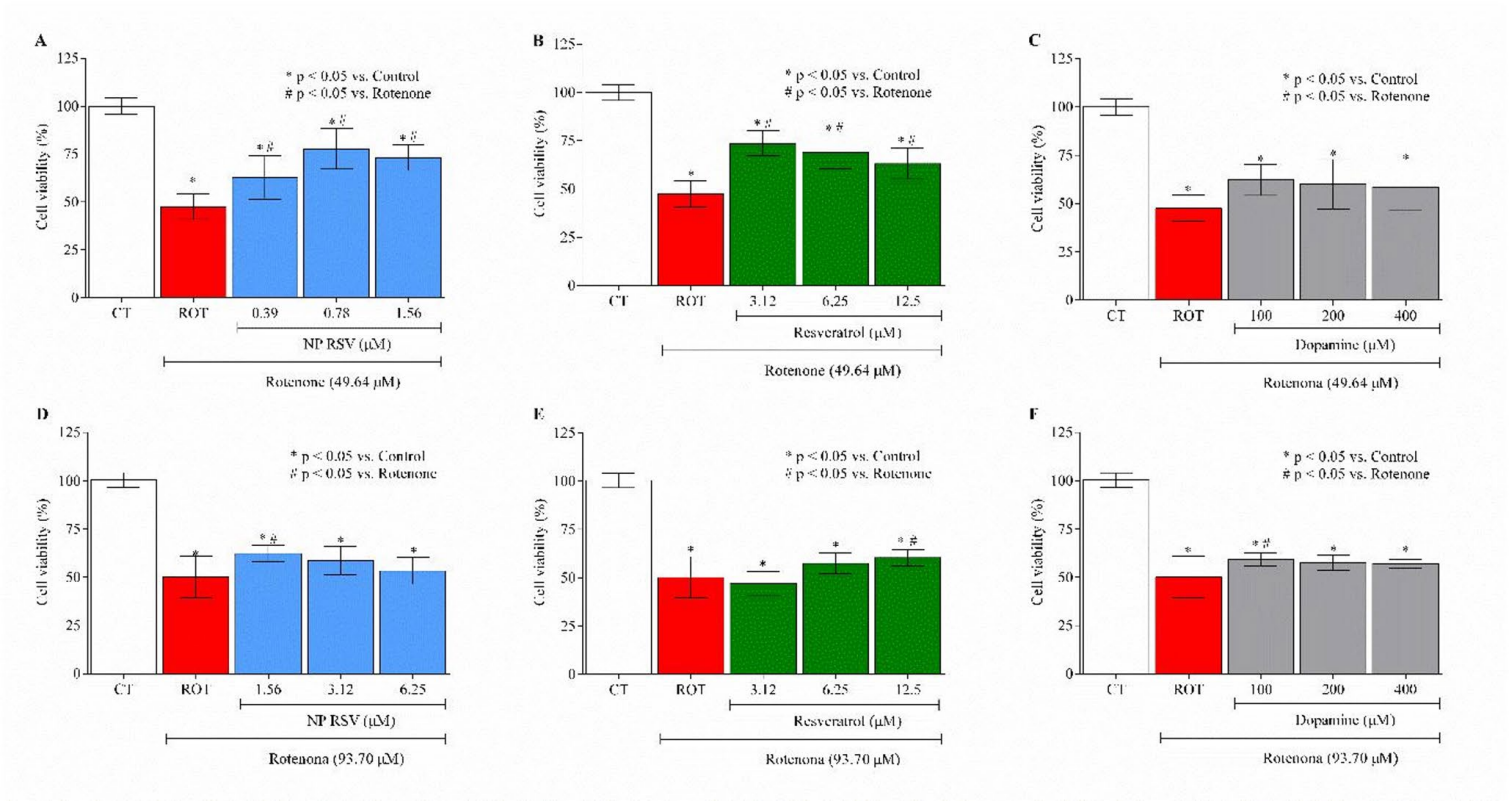
MTT reduction cell viability assay showing NP RSV protection in PC12 and AST cell lines after exposure to ROT (*n* = 3). **PC12**: **A** NP RSV + ROT; **B** RSV + ROT; **C** DA + ROT; **AST**: **D** NP RSV + ROT; **E** RSV + ROT; **F** DA + ROT. Results are shown as mean ± SD. *p* < 0.05 compared to control. For statistical analysis, one-way ANOVA was used, followed by Bonferroni post-test, CT = Negative control; ROT = Rotenone; *p < 0.05 compared to the control group; ^#^*p* < 0.05 compared to the ROT group. All p values obtained were < 0.0001. F value: PC12—**A** (83.19); **B** (112.3); **C** (147.7); AST—**D** (37.52); **E** (55.36); **F** (35.24). This figure was generated using GraphPad Prism 8 software

Subsequently, a test was conducted to evaluate the protective potential of NP RSV, comparing it with RSV and DA after lesion induction by ROT (IC_50_: 49.64 µM - PC12; 93.70 µM - AST). The results obtained in PC12 showed a protective effect, especially at a concentration of 0.78 µM NP RSV (Fig. 1A), partially restoring cell viability compared with ROT-treated cells (77.66%). The most protective concentration of RSV was 3.12 µM, with a viability of 73.66% (Fig. 1B). Lower concentrations of NP RSV showed a similar protective effect. In AST, only the concentration of 1.56 µM NP RSV (Fig. 1D) was protective (viability of 62.33%), while RSV showed protection at 12.5 µM (viability of 60.33%) (Fig. 1D). In contrast, DA only provided protection at a concentration of 100 µM (Fig. 1F) in AST, with cell viability equal to 59.33%.

### Evaluation of Cell Death Pathways by Flow Cytometry

**Fig. 2:**
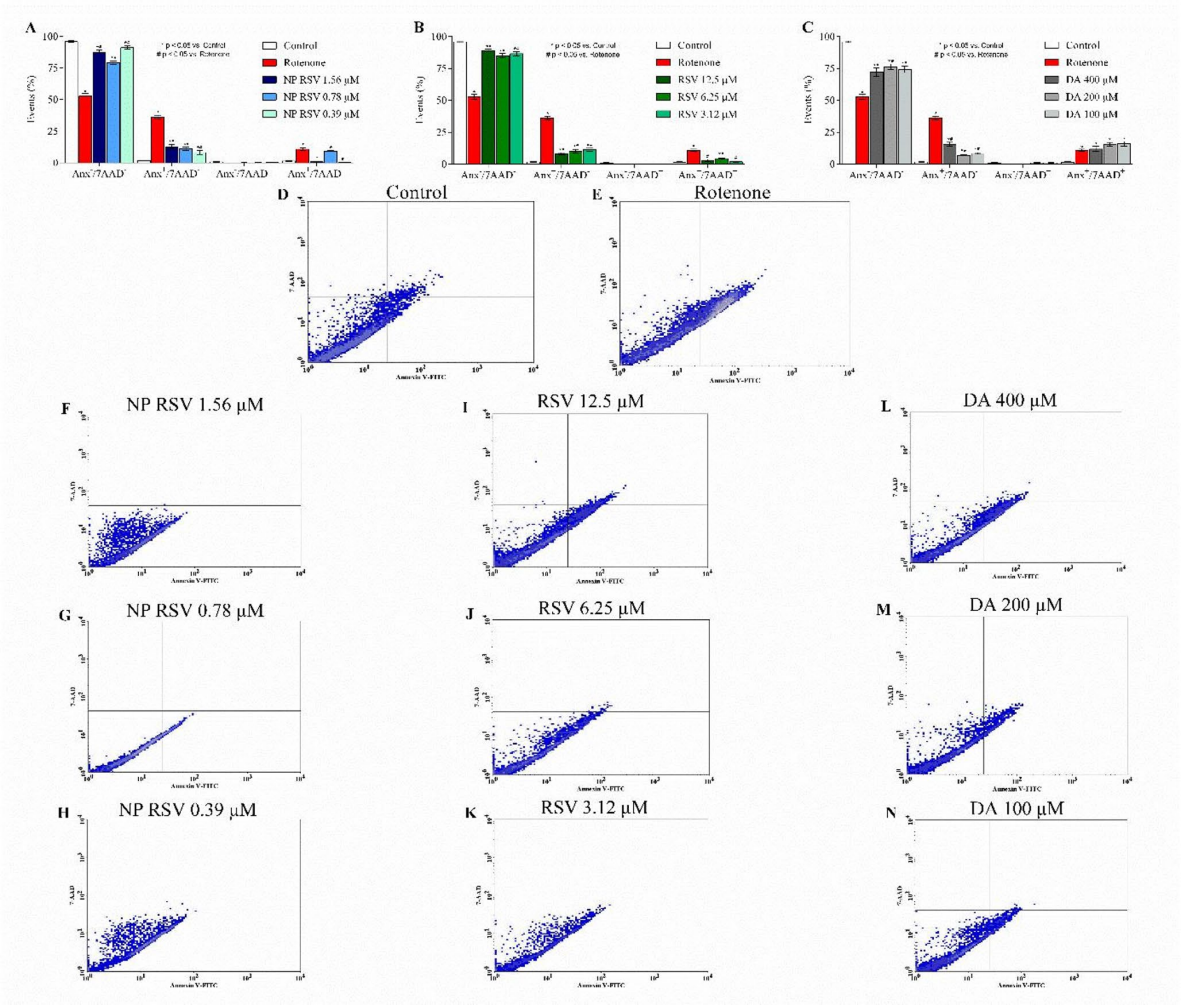
Representation of the cell death pathway assessment assay in PC12. Necrotic cells marked by 7AAD (7-aminoactinomycin) and apoptotic cells marked by Anx (Annexin-V). Y-axis: Necrotic cells marked by 7AAD (7-aminoactinomycin) and X-axis: apoptotic cells marked by Anx (annexin V). Results are shown as mean ± SD. *p* < 0.05 compared to control. For statistical analysis, two-way ANOVA was used, followed by Bonferroni post-test; *p < 0.05 compared to control group; ^#^*p* < 0.05 compared to ROT group. All p values obtained were < 0.0001. This figure was generated using GraphPad Prism 8 software and Cell Quest Pro™ software

**Fig. 3:**
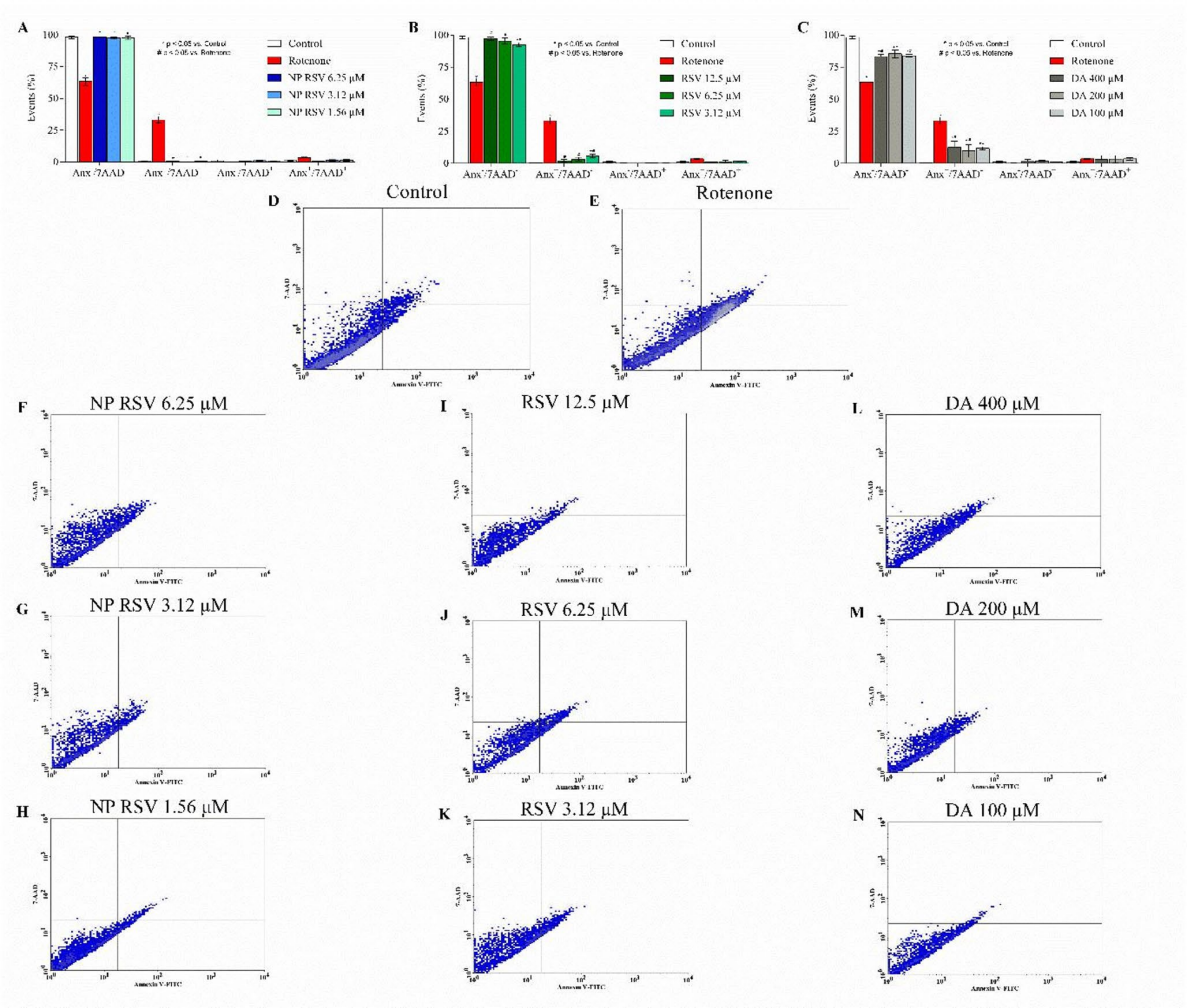
Representation of the cell death pathway assessment assay in AST. Necrotic cells marked by 7AAD (7-aminoactinomycin) and apoptotic cells marked by Anx (annexin V). Y-axis: Necrotic cells marked by 7AAD (7-aminoactinomycin) and X-axis: apoptotic cells marked by Anx (annexin V). Results are shown as mean ± SD. *p* < 0.05 compared to control. For statistical analysis, two-way ANOVA was used, followed by Bonferroni post-test; *p < 0.05 compared to control group; ^#^*p* < 0.05 compared to ROT group. All p values obtained were < 0.0001. This figure was generated using GraphPad Prism 8 software and Cell Quest Pro™ software

To evaluate the effect of NP RSV on ROT-induced cell death pathways, a flow cytometry assay was performed using 7AAD/Annexin V-PE labeling (Figs. 2, 3). Both PC12 (36.24% of events) and AST (33.16% of events) showed significant labeling with Annexin-V (7AAD−/Anx+) when exposed to ROT when compared to the control, indicating early apoptosis. In addition, a population of double-labeled cells (7AAD+/Anx+) was present in the ROT group of PC12 cells, indicating a process suggestive of late apoptosis or secondary necrosis (10.85% of events).

NP RSV significantly reduced apoptosis in PC12 cells. Treatment with 1.56, 0.78, and 0.39 µM decreased the Annexin V-positive population by 23.56%, 25.07%, and 28%, respectively (Fig. 2). In the double-labeled cell population, there was a reduction of 10.01% and 10.56% at concentrations of 1.56 and 0.39 µM, respectively. The same was observed in AST (Fig. 3), where there was a reduction of 32.54%, 33.01%, and 32.74% of events in the groups treated with NP RSV 6.25, 3.12, and 1.56 µM, respectively. The density plot are shown in Figs. 2F−N and 3F−N.

As for RSV, it was protective in the three concentrations used and reduced Annexin V-labeled and double-labeled events in both cell lines. DA was also able to protect cells, reducing the percentage of Annexin V-labeled events, but did not reduce double-labeled events in PC12 cells.

### Analysis of Cytoplasmic ROS Production

**Fig. 4:**
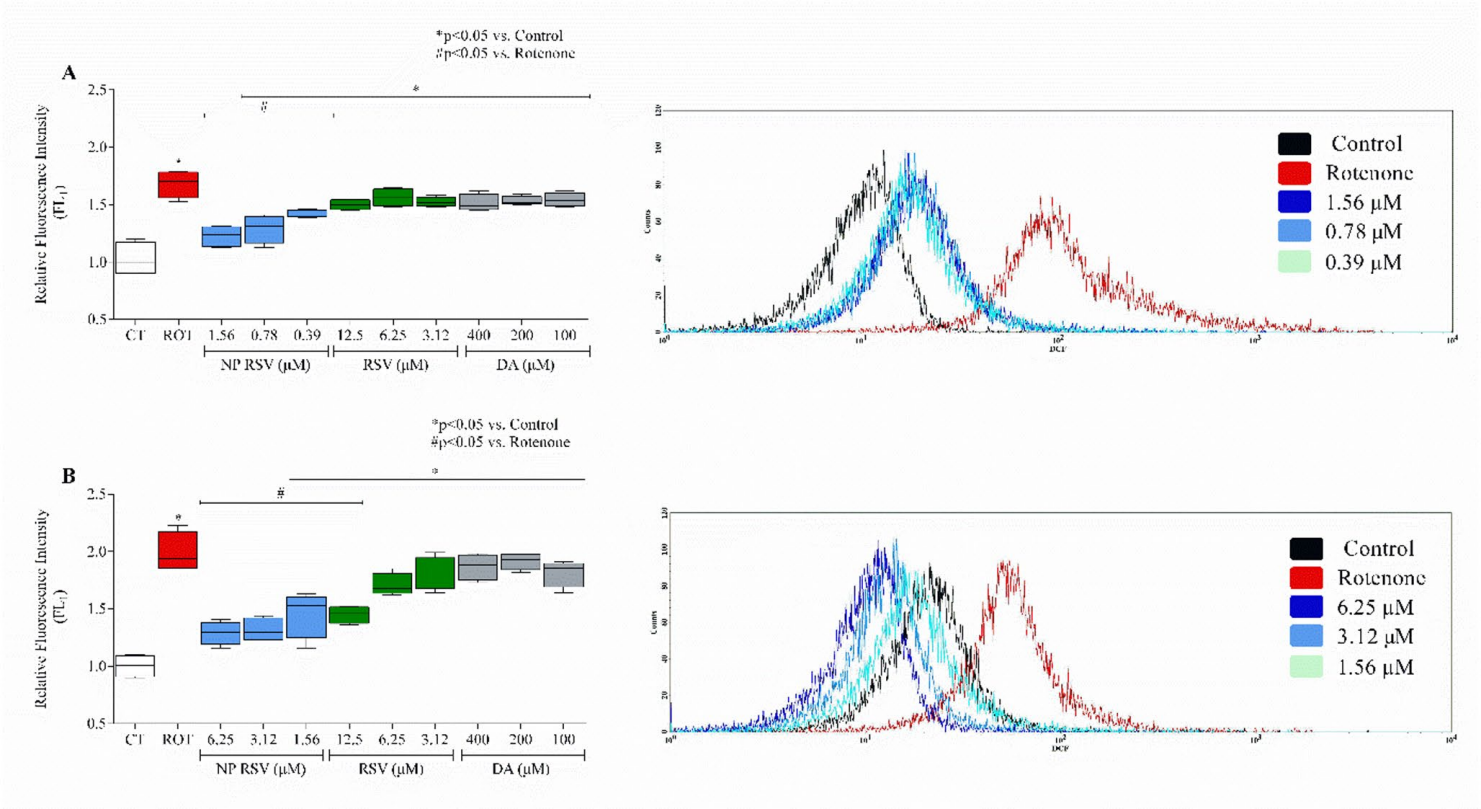
Analysis of oxidative stress and cytoplasmic ROS production (FL_1_) in PC12 (**A**) and AST (**B**) by flow cytometry using the DCFH-DA assay. Data are expressed as fluorescence relative to control ± SD. *p* < 0.05. Data were analyzed by one-way ANOVA with Bonferroni post-test. *p < 0.05 compared to the control group; ^#^*p* < 0.05 compared to the ROT group. Representative histogram of the DCFH-DA assay, showing the group treated with NP RSV at concentrations of 1.56 µM, 0.78 µM, and 0.39 µM in PC12 and 6.25 µM, 3.12 µM, and 1.56 µM in AST. All p-values obtained were < 0.0001. F value: **A** 19.05; **B** 25.33. This figure was generated using GraphPad Prism 8 software and Cell Quest Pro™ software

Exposure to ROT significantly increased ROS production by 168% in PC12 (Fig. 4A). In addition, NP RSV treatment reduced ROS levels in PC12 by 46%, 39%, and 26% at concentrations of 1.56, 0.78, and 0.39 µM, respectively. The RSV and DA groups did not significantly reduce ROS levels.

ROT increased ROS production in AST by 198% (Fig. 4B), in terms of relative fluorescence intensity. Pre-treatment with NP RSV reduced this increase by 68% in the NP RSV at 6.25 µM, 67% in the NP RSV at 3.12 µM, and 52% in the NP RSV at 1.56 µM, while RSV at 12.5 µM reduced it by 53%. There was no significant change in the DA group. Representative histograms of cell populations are shown in Fig. 4. The values ​​are shown in Table 2 of the supplementary material.

### Assessment of Mitochondrial Transmembrane Potential (ΔΨm)

**Fig. 5:**
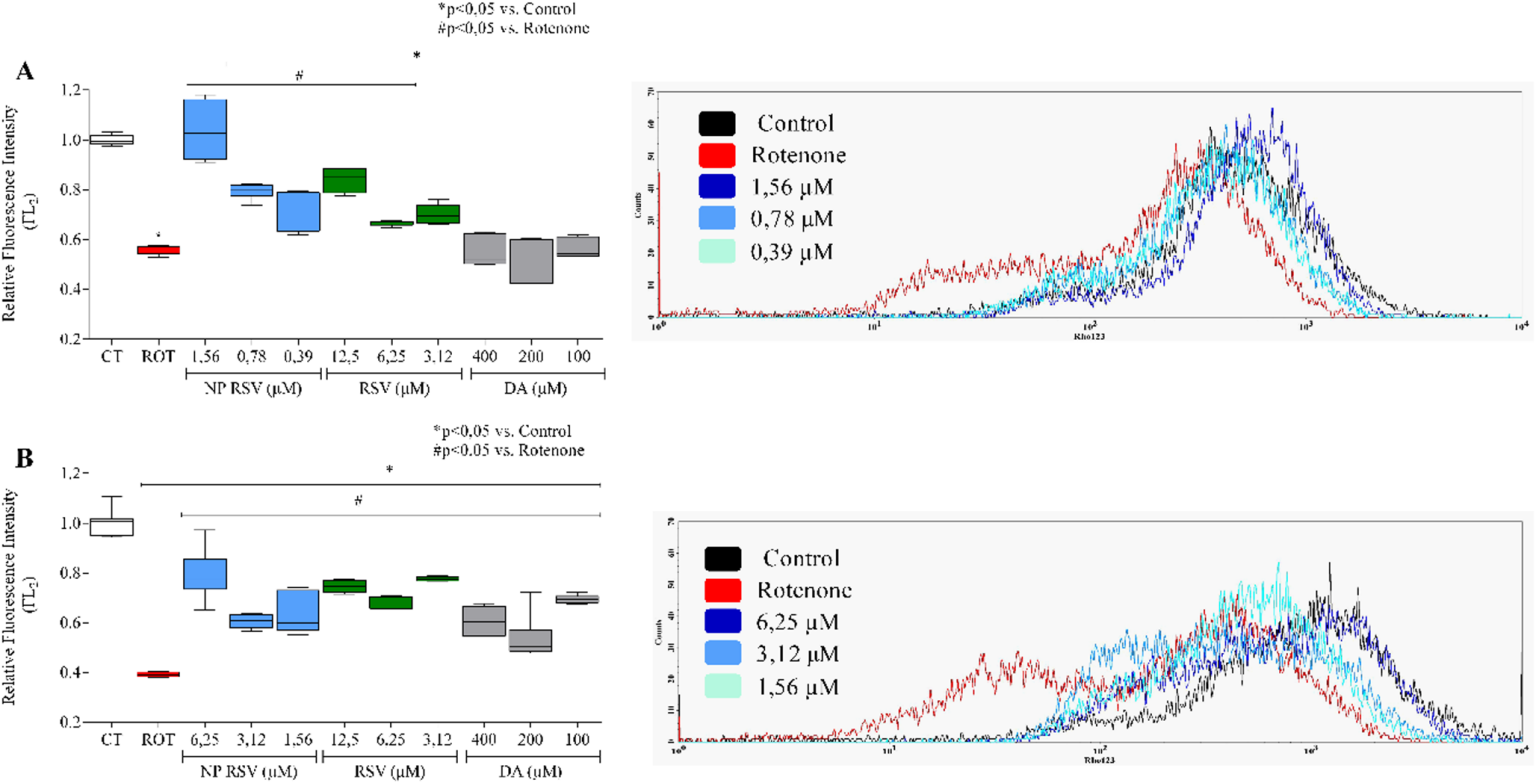
Analysis of mitochondrial transmembrane potential (FL_2_) in PC12 (**A**) and AST (**B**) cells by flow cytometry using the Rhodamine 123 (Rho 123) assay. Data are expressed as relative fluorescence to control ± SD. *p* < 0.05. Data were analyzed by one-way ANOVA with Bonferroni post-test. *p < 0.05 compared to the control group; #*p* < 0.05 compared to the ROT group. Representative histogram of the Rho123 assay, showing the NP RSV-treated group in PC12 (**A**) and AST (**B**). All p values obtained were < 0.0001. F value: **A** 60.30; **B** 54.32. This figure was generated using GraphPad Prism 8 software and Cell Quest Pro™ software

Cells exposed to ROT had approximately only half of the fluorescence related to the evaluation of the potential compared to the control group in both cell lines, indicated by the decrease in Rho-123 labeling. In PC12, NP RSV partially restored mitochondrial membrane potential, with the greatest effect observed at 1.56 µM (Fig. 5A), with a 3.7% increase in ΔΨm compared to the control group. In addition, all other NP RSV concentrations showed protection, while RSV at 6.25 µM and all tested concentrations of DA did not show protection of ΔΨm.

A slightly different response pattern was observed in AST cells., in AST cells (Fig. 5B), all other NP RSV concentrations provided protection, as did all concentrations of RSV and DA. NP RSV partially restored mitochondrial membrane potential. NP RSV at 1.56 µM showed the best result, as it increased ΔΨm by 202.19% compared to the ROT group. A representative histogram of both cell populations is shown in Fig. 5. The values ​​are shown in Table 3 of the supplementary material.

### Optical Microscopy

**Fig. 6:**
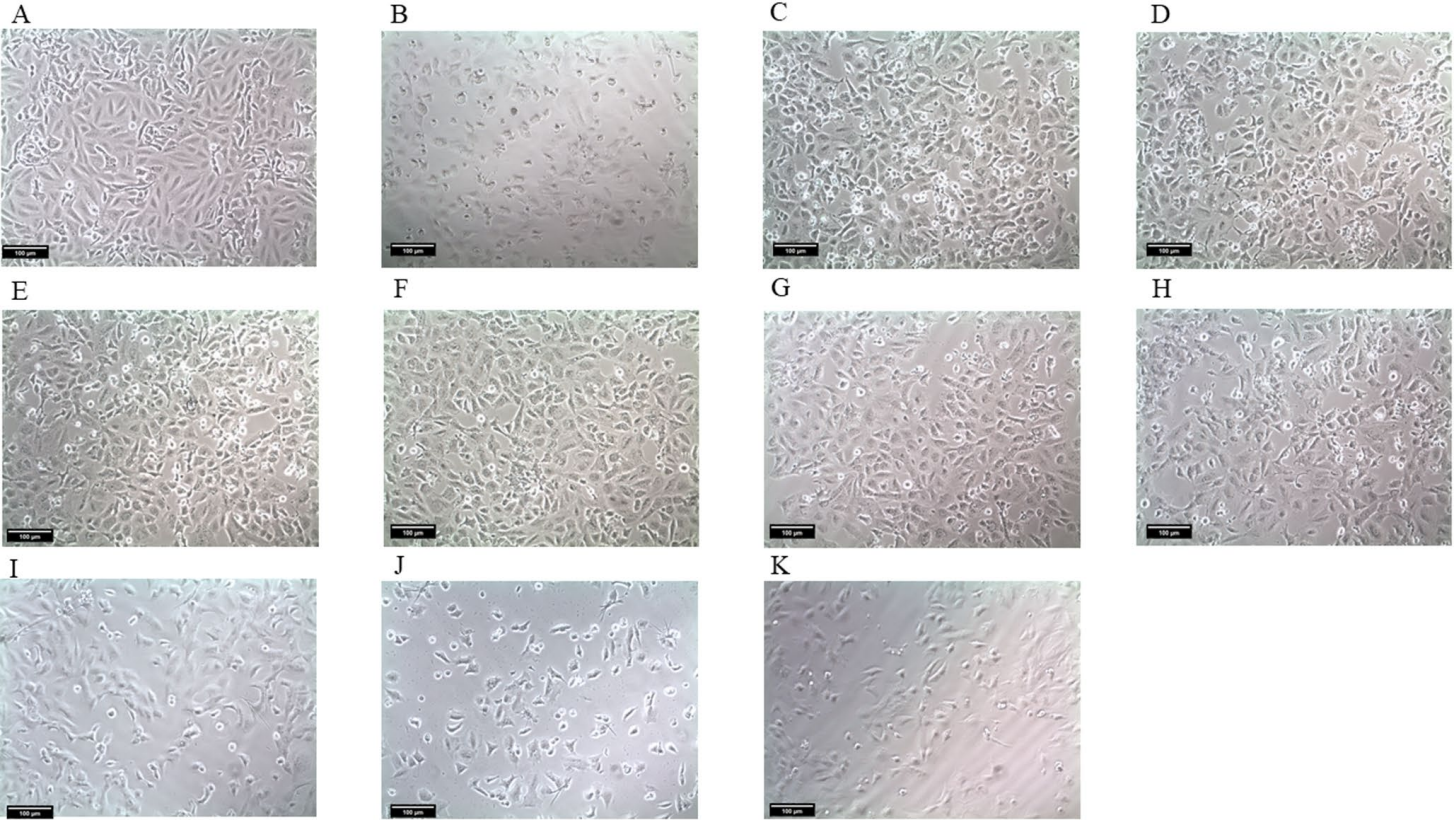
Optical microscopy images at ×100 magnification of PC12 cells. **A** Control group; **B** Rotenone; **C** NP RSV 1.56 µM; **D** NP RSV 0.78 µM; **E** NP RSV 0.39 µM; **F** RSV 12.5 µM; **G** RSV 6.25 µM; **H** RSV 3.12 µM; **I** DA 400 µM; **J** DA 200 µM; **K** DA 100 µM. Images generated by Infinity Analyze software

**Fig. 7:**
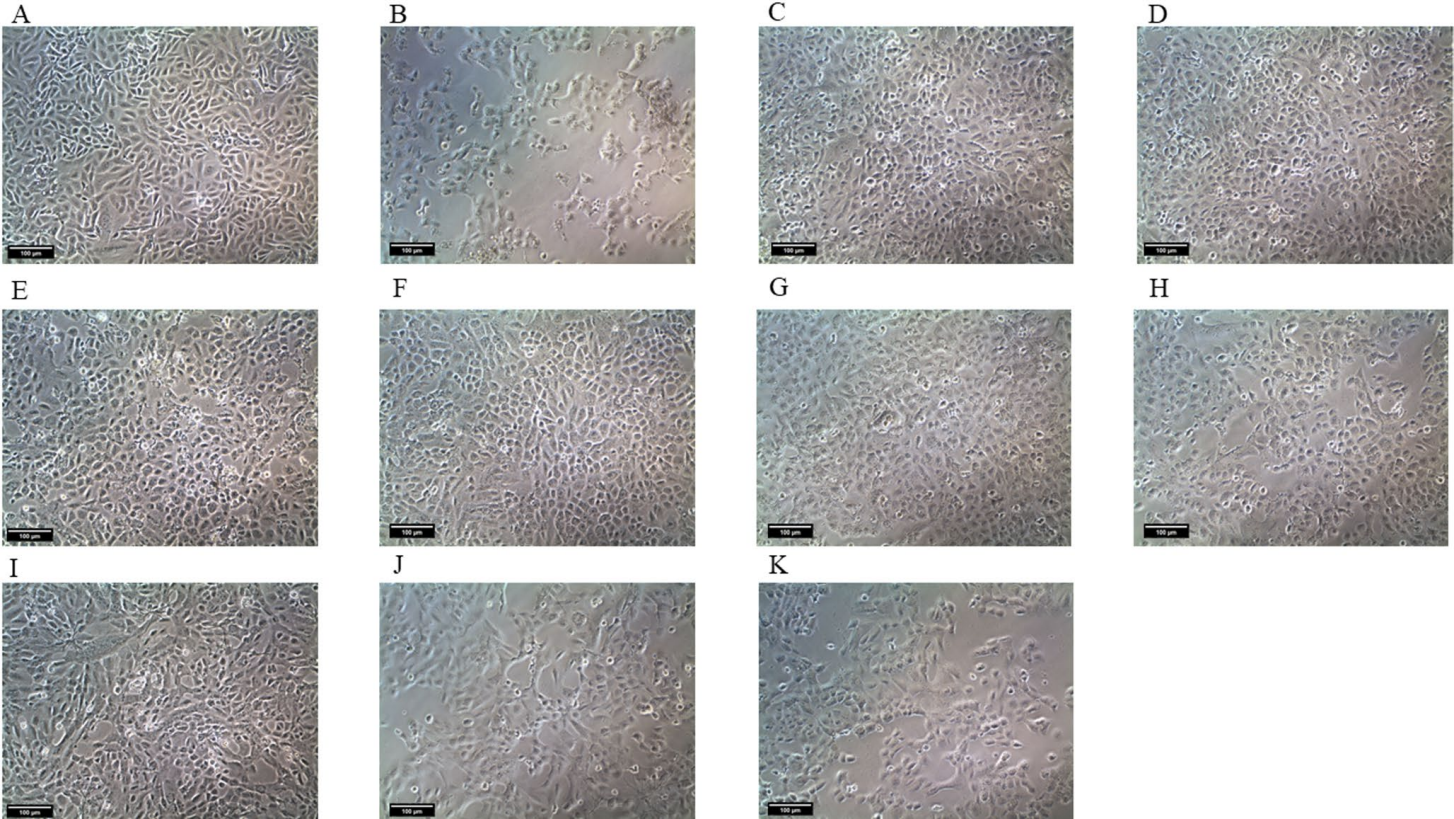
Optical microscopy images at ×100 magnification of AST. **A** Control group; **B** Rotenone; **C** NP RSV 6.25 µM; **D** NP RSV 3.12 µM; **E** NP RSV 1.56 µM; **F** RSV 12.5 µM; **G** RSV 6.25 µM; **H** RSV 3.12 µM; **I** DA 400 µM; **J** DA 200 µM; **K** DA 100 µM. Images generated by Infinity Analyze software

Cells were analyzed by optical microscopy to observe qualitative cellular changes for PC12 (Fig. 6) and AST (Fig. 7). Normal cells were used as controls (Figs. 6A, 7A), while cells subjected to the ROT injury protocol showed morphological changes (Figs. 6B, 7B), such as cell volume shrinkage and decreased cell density. Treatments with NP RSV at concentrations of 1.56 µM, 0.78 µM, and 0.39 µM for PC12 (Fig. 6C−E) and 6.25 µM, 3.12 µM, and 1.56 µM for AST (Fig. 7C−E) were able to partially protect and maintain cell morphology, as well as preserve cell density. RSV at the tested concentrations was also able to partially maintain cell morphology and density (Figs. 6F−H, 7F−H), as was DA (Figs. 6I−K, 7I−K), although to a lesser extent than NP RSV. All images were captured at 100x magnification using an inverted optical microscope.

### Gene Expression Assay

**Fig. 8:**
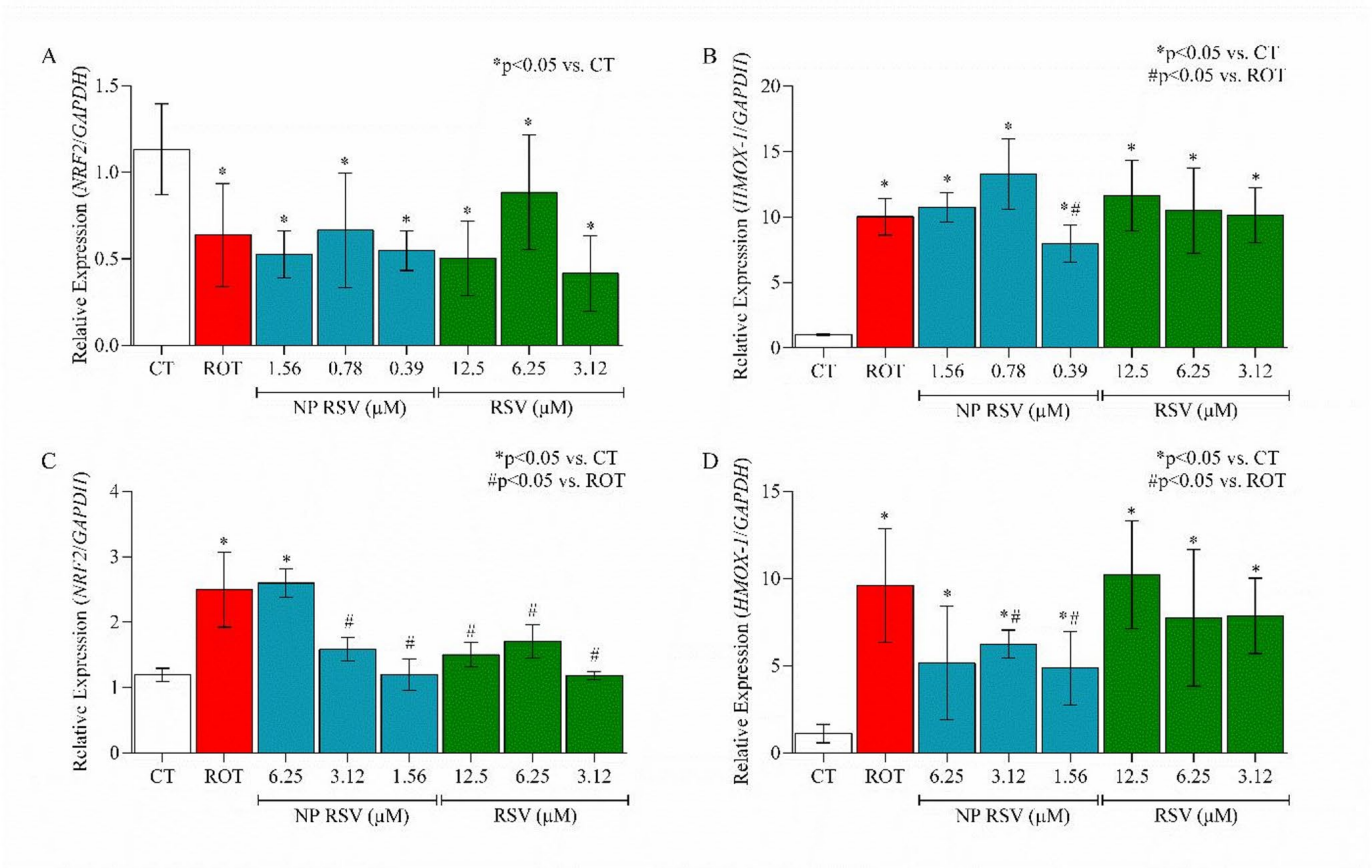
**A**,** B** Relative expression of *NRF2*/*GAPDH* and *HMOX-1*/*GAPDH* mRNA in PC12 cells. **C**,** D** Relative expression of *NRF2*/*GAPDH* and *HMOX-1*/*GAPDH* mRNA in AST. All p values obtained were < 0.0001. F value: **A** 7.250; **B** 24.74; **C** 7.431; **D** 9.787. This figure was generated using GraphPad Prism 8 software

To evaluate the effect of NP RSV on the gene expression of *NRF2* and HMOX-1, RT-qPCR was performed. Exposure to ROT significantly altered the expression of both genes in the two cell lines studied. A stronger modulation of NRF2 expression was observed in AST cells, particularly with NP RSV at 3.12 µM, which reduced relative expression by 36.5%. All RSV concentrations were able to reduce relative expression (Fig. 8C) compared to ROT, with relative expression results close to those of the control. In PC12 cells, this modulation could not be observed (Fig. 8A).

In relation to HMOX-1, a significant decrease in relative expression was observed in PC12 in the NP RSV at 0.39 µM, with a reduction of 20.6% compared to ROT (Fig. 8B). In AST, there was a reduction of 34.9% in the NP RSV at 3.12 µM and 50% in the NP RSV 1.56 µM compared to cells exposed to ROT (Fig. 8D), indicating protection against ROS in these pretreated groups. The Ct and relative fold-change values ​​are shown in Table 3 of the supplementary material.

## Discussion

PD remains a major therapeutic challenge because current pharmacological approaches, such as l-Dopa, primarily alleviate symptoms without preventing the progression of neurodegeneration [39]. Considering that oxidative stress and mitochondrial dysfunction play a central role in the pathophysiology of PD, it is essential to investigate therapeutic strategies capable of modulating endogenous antioxidant pathways [40].

In this context, RSV has stood out for its antioxidant and neuroprotective properties, especially for its ability to modulate the NRF2/Keap1/ARE pathway, one of the main cellular defense systems against oxidative stress, responsible for inducing antioxidant and cytoprotective genes [8]. However, its therapeutic application is limited by pharmacokinetic disadvantages, such as short half-life, low water solubility, and reduced bioavailability in vivo, limiting its ability to cross the blood–brain barrier (BBB) [15]. Given these limitations, the nanoencapsulation of RSV emerges as a promising strategy to enhance its efficacy. Therefore, the present study investigated the neuroprotective effect of NP RSV in an in vitro model of ROT-induced PD.

In addition, the characterization and functionalization of these polymeric NPs were performed by Cavalcante de Freitas et al. (2023), where the results obtained also suggest the development of formulations with physicochemical properties suitable for the intended therapeutic application. The PCL-based composition with the nonionic surfactant TPGS demonstrated good biocompatibility with the active ingredient [15]. TPGS has already been reported in the literature as an important factor in prolonging drug circulation, improving the release of the encapsulated drug, and increasing encapsulation efficiency, as well as reducing drug resistance [20–22].

The results of the study demonstrated that NP RSV exerted a significant cytoprotective effect on neuronal cells and astrocytes exposed to ROT, being effective in reducing oxidative stress, preserving mitochondrial membrane potential, and decreasing apoptosis. These effects were observed at concentrations lower than those required for RSV, indicating an enhancement of pharmacological efficacy resulting from nanoencapsulation. Considering that ROT promotes neurotoxicity mainly by inhibiting mitochondrial complex I and consequently increasing the production of ROS, the data suggest that NP RSV act efficiently in preserving mitochondrial homeostasis [41].

In PD, dopaminergic neuronal loss occurs predominantly through apoptosis, usually mediated by mitochondrial dysfunction. However, in situations involving intense or prolonged exposure to damage, necrosis and other mechanisms of cell death occur [40]. In the present study, NP RSV significantly reduced ROT-induced apoptotic populations. Studies indicate that RSV is capable of attenuating mitochondrial dysfunction and cell apoptosis. These effects are related to its activity on Akt/glycogen synthase kinase-3β (GSK-3β) protein, through the inhibition of GSK-3β activity by Akt, since GSK-3β is crucial in cellular functions such as apoptosis, cell signaling, and proliferation [42]. In addition, it regulates Bcl-2 expression and inhibits the activation of initiator and executor caspases, such as caspase-9 and caspase-3 [43, 44].

In the present study, mitochondrial depolarization was observed due to a reduction in ΔΨm induced by ROT. This effect is directly associated with the permeabilization of the outer mitochondrial membrane and the release of pro-apoptotic factors, such as cytochrome C, culminating in the activation of executor caspases and an increase in apoptotic cell populations detected by Anx-V [43, 44].

The effect of RSV is linked to mitochondrial bioenergetic modulation. It acts by preserving ΔΨm, reducing membrane permeabilization, and inhibiting the release of cytochrome C from the mitochondrial intermembrane space. In addition, it also modulates complex I by activating sirtuin 1 (SIRT1), AMPK, PGC-1α, and NRF2/HMOX-1, playing a role in immune modulation by binding to Toll-like receptors (TLR) 4 [9, 13, 45, 46].

Due to mitochondrial dysfunction caused by ROT, there was a significant increase in ROS. This is observed by the increase in DCF labeling in cells exposed to ROT. Pretreatment with NP RSV was able to significantly reduce ROS production, preserving ΔΨm. These findings corroborate the potential of NP RSV in reducing apoptotic events.

NP RSV act in an integrated manner to maintain mitochondrial homeostasis, with the NRF2/HMOX-1 pathway playing a key role. The induction of *HMOX-1* has been associated with reduced ROS, mitochondrial stabilization, and anti-apoptotic effects in models of ROT-induced neurotoxicity, reinforcing the hypothesis that the action of NP RSV involves not only the direct neutralization of reactive species but also the activation of endogenous cytoprotective responses [47–49].

Molecular biology assays revealed different effects on the NRF2/Keap1/ARE pathway in the two cell lines studied. PC12 cells, being neuronal cells, did not show significant results in *NRF2* expression, while in AST, ROT increased the relative expression of this gene, and NP RSV acted in modulation, maintaining results closer to the control group. This is related to the function of this cell, since AST participate in CNS homeostasis and defense against oxidative stress, presenting higher basal expression of *NRF2* and greater capacity for activation of ARE genes, which are fundamental for the expression of *HMOX-1*. This role of AST is fundamental for the protection of neurons. In contrast, dopaminergic neurons are directly affected, leading to death by oxidative factors [50].

Regarding HMOX-1, it was more highly expressed in cells exposed to ROT, as they required antioxidant defense mechanisms, and NP RSV maintained lower levels of expression of these genes in both lines. Heme oxygenase is an *NRF2*-adjuvant effector protein responsible for the degradation of the heme group into ferrous ion (Fe²⁺), carbon monoxide, and biliverdin, and it is a key enzyme in the prevention of oxidation [8, 49, 51].

Neuropathological studies show increased expression of *HMOX-1* in the substantia nigra of Parkinson’s patients, which may be an adaptive response to excessive ROS production resulting from mitochondrial dysfunction [9, 52]. However, this endogenous response is often insufficient to contain the progression of neurodegeneration, making pharmacological modulation of *HMOX-1* a target of therapeutic interest [53].

The neuroprotection observed may involve modulation of the NRF2/HMOX-1 pathway, since this regulates antioxidant and cytoprotective mechanisms essential for maintaining mitochondrial homeostasis and reducing apoptotic processes induced by oxidative stress [8, 54]. In this sense, exposure to ROT promoted a significant increase in the production of ROS and mitochondrial depolarization, events directly related to the activation of pro-apoptotic pathways. In contrast, pretreatment with NP RSV significantly reduced the generation of these species, preserved mitochondrial membrane potential, and decreased apoptotic cells, indicating the inhibition of mitochondrial events involved in programmed cell death [44]. Together, these findings support the hypothesis that redox homeostasis is restored, with the NRF2 pathway playing a central role in the neuroprotection observed.

Furthermore, the NRF2/Keap1/ARE pathway is one of the main cellular defense systems against oxidative stress, regulating the expression of antioxidant enzymes, including HMOX-1, a direct transcriptional target of the NRF2 factor. This enzyme plays a key role in reducing reactive oxygen species, stabilizing mitochondria, and modulating anti-apoptotic mechanisms, and it is considered a functional marker of the activation of this pathway [8]. Neuropathological studies indicate increased expression of heme oxygenase-1 in the substantia nigra of PD patients, possibly as an adaptive response to chronic oxidative stress, although this endogenous response is often insufficient to contain the progression of neurodegeneration [53]. In this scenario, pharmacological modulation of this pathway acts as a promising therapeutic target.

RSV has been reported as a modulator of the NRF2/HMOX-1 pathway [15]. Previous studies corroborate the findings of the present work, demonstrating that NP RSV have a greater capacity to reduce oxidative stress, preserve mitochondrial function, and exert anti-neuroinflammatory effects when compared to the isolated compound [55].

Although this study has limitations inherent to in vitro models, such as the absence of bioavailability assessment, half-life, and direct protein expression, the results indicate that NP RSV exerts a relevant neuroprotective effect in a cellular model of PD induced by ROT. Taken together, the data suggest that resveratrol nanoencapsulation enhances its ability to modulate the NRF2/Keap1/ARE pathway, resulting in reduced oxidative stress, preservation of mitochondrial function, and attenuation of neuronal apoptosis. In this context, these findings reinforce the potential of nanotechnology-based strategies as promising approaches for the prevention and management of PD-associated neurodegeneration, justifying further in vivo studies and additional investigations aimed at future clinical application.

## Conclusion

The results demonstrated that NP RSV exert a neuroprotective effect on PC12 cells and astrocytes against damage caused by ROT in an in vitro model mimicking PD. This effect is associated with modulation of the Keap1/NRF2/ARE pathway, possibly related to modulation of the Keap1/NRF2/ARE pathway, promoting protection against oxidative stress.

Activation of this pathway resulted in preservation of mitochondrial function, reduction of apoptosis, and increased cell viability. HMOX-1, as a relevant transcriptional target of the Keap1/NRF2/ARE pathway, stood out as an important mediator of the cytoprotective effects observed. Together, the data indicate that NP RSV represent a promising strategy for modulating antioxidant and mitochondrial mechanisms involved in the pathophysiology of PD.

## Electronic Supplementary Material

Below is the link to the electronic supplementary material.

Supplementary Material 1

## Data Availability

No datasets were generated or analysed during the current study.
