# Supplementary material for: Resveratrol-Loaded Polymeric Nanoparticles Protect Against Rotenone-Induced Parkinsonian-Like Cellular Damage In Vitro: Association with NRF2/HMOX-1 Expression Changes
Source: Neurochem Res. 2026 Apr 13;51(2):138. doi: 10.1007/s11064-026-04749-z (PMC13076504; doi:10.1007/s11064-026-04749-z)
Supplement: Supplementary file 1 — Supplementary Material 1 [file 11064_2026_4749_MOESM1_ESM.docx]

**SUPPLEMENTARY MATERIAL**





**Fig. 9** Cell viability graphs in PC12 cells and AST. A – resveratrol polymeric nanoparticles (µM) in PC12; B – nanoparticles without resveratrol (µM) in PC12; C – resveratrol (µM) in PC12; D – dopamine (µM) in PC12; E – resveratrol polymeric nanoparticles (µM) in AST; F – nanoparticles without resveratrol (µM) in AST; G – resveratrol (µM) in AST; H – dopamine (µM) in AST. Results are shown as mean ± SD. p <0.05 compared to control groups. For statistical analysis, one-way ANOVA was used, followed by the Bonferroni post-test. All p-values obtained were < 0.0001. F value: PC12 - A (39.77); B (0,9231); C (20.49) D (6.833); AST - E (75.55); F (36.99); G (25.55); H (3.731). CT = Negative control, V = Vehicle (DMSO); *p < 0.05.

| Cell line | Events | RFI±SD |
| --- | --- | --- |
| PC12 | Control | 1.00±0.15 |
|  | Rotenone | 1.68±0.11* |
|  | NP RSV 1.56 µM | 1.22±0.09# |
|  | NP RSV 0.78 µM | 1.29±0.12*# |
|  | NP RSV 0.39 µM | 1.42±0.02*# |
|  | RSV 12.5 µM | 1.5±0.04* |
|  | RSV 6.25 µM | 1.56±0.7* |
|  | RSV 3.12 µM | 1.52±0.04* |
|  | DA 400 µM | 1.51±0.07* |
|  | DA 200 µM | 1.53±0.04* |
|  | DA 100 µM | 1.54±0.06* |
| AST | Control | 1.00±0.09 |
|  | Rotenone | 1.98±0.17* |
|  | NP RSV 6.25 µM | 1.29±0.10# |
|  | NP RSV 3.12 µM | 1.31±0.10# |
|  | NP RSV 1.56 µM | 1.46*±0.20# |
|  | RSV 12.5 µM | 1.45±0.07*# |
|  | RSV 6.25 µM | 1.70±0.09* |
|  | RSV 3.12 µM | 1.80±0.14* |
|  | DA 400 µM | 1.86±0.11* |
|  | DA 200 µM | 1.91±0.06* |
|  | DA 100 µM | 1.81±0.11* |

**Table 2.** Values ​​from the evaluation of cytoplasmic ROS production. Relative fluorescence intensity (RFI) of PC12 and AST cells subjected to the ROT model and pre-treated with different concentrations of RSV NP. Data are expressed as mean ± standard deviation (SD) and evaluated by one-way ANOVA with Bonferroni post-test. p<0.05. CT = Negative control; ROT = Rotenone; NP RSV = Resveratrol polymeric nanoparticles; RSV = Resveratrol. *p<0.05 compared to the control group; #p<0.05 compared to the ROT group.

| Cell line | Events | RFI±SD |
| --- | --- | --- |
| PC12 | Control | 1.00 ± 0.00 |
|  | Rotenone | 0.55 ± 0.01* |
|  | NP RSV 1.56 µM | 1.03 ± 0.12# |
|  | NP RSV 0.78 µM | 0.79 ± 0.03*# |
|  | NP RSV 0.39 µM | 0.71 ± 0,03*# |
|  | RSV 12.5 µM | 0.84 ± 0,04*# |
|  | RSV 6.25 µM | 0.66 ± 0.01* |
|  | RSV 3.12 µM | 0.70 ± 0.04*# |
|  | DA 400 µM | 0.55 ± 0.06* |
|  | DA 200 µM | 0.48 ± 0.09* |
|  | DA 100 µM | 0.56 ± 0.04* |
| AST | Control | 1.00 ± 0.05 |
|  | Rotenone | 0.39 ± 0.00* |
|  | NP RSV 6.25 µM | 0.79 ± 0.10*# |
|  | NP RSV 3.12 µM | 0.60 ± 0.03*# |
|  | NP RSV 1.56 µM | 0.63 ± 0.08*# |
|  | RSV 12.5 µM | 0.74 ± 0.02*# |
|  | RSV 6.25 µM | 0.68 ± 0.02*# |
|  | RSV 3.12 µM | 0.77 ± 0.00*# |
|  | DA 400 µM | 0.60 ± 0.06*# |
|  | DA 200 µM | 0.53 ± 0.09*# |
|  | DA 100 µM | 0.69 ± 0.01*# |

**Table 3.** Values ​​from the assessment of mitochondrial transmembrane potential. Relative fluorescence intensity (RFI) of PC12 and AST cells subjected to the ROT model and pre-treated with different concentrations of RSV NP. Data are expressed as mean ± standard deviation (SD) and evaluated by one-way ANOVA with Bonferroni post-test. p<0.05. CT = Negative control; ROT = Rotenone; NP RSV = Resveratrol polymeric nanoparticles; RSV = Resveratrol. *p<0.05 compared to the control group; #p<0.05 compared to the ROT group.

|  |  | *NRF2/GAPDH* | | *HMOX/GAPDH* | | *GAPDH* |
| --- | --- | --- | --- | --- | --- | --- |
| Cell line |  | Ct±SD | Relative fold-change | Ct±SD | Relative fold-change | Ct±SD |
| PC 12 | CT | 25.1 ±1.44 | 1.13±0.09 | 18.75±1.51 | 1.00±0.03 | 14.14±0.48 |
|  | ROT | 26.25±0.79 | 0.64±0.11* | 17.00±0.25 | 10.0±0.50* | 14.68±0.41 |
|  | NP RSV 1.56 µM | 25.41±1.03 | 0.53±0.05* | 16.91±0.40 | 10.8±0.46* | 14.61±0.39 |
|  | NP RSV 0.78 µM | 26.37±0.39 | 0.67±0.14* | 17.19±0.34 | 13.3±0.95* | 15.07±0.67 |
|  | NP RSV 0.39 µM | 25.93±0.87 | 0.55±0.04* | 18.09±1.58 | 7.96±0.50*# | 14.57±0.28 |
|  | RSV 12.5 µM | 26.37±0.62 | 0.50±0.08* | 17.07±0.17 | 11.7±0.95* | 14.94±0.23 |
|  | RSV 6.25 M | 26.42±0.85 | 0.88±0.12* | 16.85±0.45 | 10.5±1.15* | 14.53±0.45 |
|  | RSV 3.12 µM | 26.42±0.68 | 0.42±0.08* | 16.85±0.32 | 10.2±0.74* | 14.53±0.20 |
| AST | CT | 26.68±0.70 | 1.20±0.09 | 20.71±1.44 | 1.12±0.19 | 15.33±0.93 |
|  | ROT | 25.62±1.17 | 2.50±0.23* | 16.64±1.45 | 9.61±1.51* | 15.41±1.00 |
|  | NP RSV 6.25 µM | 26.97±2.14 | 2.60±0.27* | 20.14±2.28 | 5.16±1.33* | 16.39±1.14 |
|  | NP RSV 3.12 µM | 26.16±0.44 | 1.59±0.18# | 18.96±1.07 | 6.25±0.33*# | 15.36±0.41 |
|  | NP RSV 1.56 µM | 26.83±0.69 | 1.20±0.24# | 19.91±1.31 | 4.88±0.75*# | 15.98±1.65 |
|  | RSV 12.5 µM | 26.71±0.34 | 1.51±0.19# | 18.90±0.93 | 10.2±1.09* | 15.88±0.76 |
|  | RSV 6.25 µM | 26.31±0.26 | 1.71±0.26# | 18.89±0.46 | 7.75±1.38* | 15.57±0.49 |
|  | RSV 3.12 µM | 26.94±0.35 | 1.19±0.06# | 19.22±0.53 | 7.87±0.68* | 15.69±0.30 |

**Table 4.** Cycle Threshold (Ct) and fold-change values ​​(exact numbers) for *NRF2*/*GAPDH*, H*MOX-1/GAPDH*, and *GAPDH* genes. The data are expressed as a relative expression ± standard deviation (SD) p<0.05. CT = Negative control; ROT = Rotenone; NP RSV = Resveratrol polymeric nanoparticles; RSV = Resveratrol. *p<0.05 compared to the control group; #p<0.05 compared to the ROT group.
